# Coffee Consumption and Risk of Gastric Cancer: A Large Updated Meta-Analysis of Prospective Studies

**DOI:** 10.3390/nu6093734

**Published:** 2014-09-18

**Authors:** Feiyue Xie, Dan Wang, Zhifang Huang, Yajun Guo

**Affiliations:** 1 The Key Lab, Cancer Center, Chinese PLA General Hospital, Beijing 100853, China; E-Mail: guoyjcc@163.com; 2 South II Department, The General Hospital of Chinese People’s Armed Police Forces, Beijing 100039, China; E-Mail: wang_dan999@126.com; 3 Department of Rheumatology and Nephrology, Air Force General Hospital, Beijing 100142, China; E-Mail: huangzf_med@126.com

**Keywords:** coffee, gastric cancer, cohort study, meta-analysis

## Abstract

The potential role of coffee consumption in the development of various types of cancer has been extensively investigated in epidemiologic studies. How coffee consumption may modulate risk of gastric cancer, however, remains a subject open for investigation. To better quantify this relation, we quantitatively summarized evidence from prospective studies. Eligible studies were identified on PubMed databases. The summary risk estimates were obtained using the random-effects model. Subgroup, sensitivity and dose-response analyses were conducted. The present meta-analysis included 12 prospective cohort studies. A pooled analysis of these studies suggested that coffee consumption (highest *vs.* lowest consumption) was not associated with risk of gastric cancer (RR = 1.12, 95% CI = 0.93–1.36). In the subgroup analysis, significant increased risk was detected in the U.S. studies (RR = 1.36, 95% CI = 1.06–1.74) and in the studies with <10 years of follow-up (RR = 1.24, 95% CI = 1.00–1.54), and the greatest increase in risk was observed in those studies without adjustment for smoking (RR = 1.48, 95% CI = 1.13–1.93). There was some evidence of publication bias (*P* for Egger’s test = 0.03). Cumulative evidence from prospective studies suggests that coffee consumption is not associated with risk of gastric cancer. The observed positive results may be confounded by smoking and need further investigation.

## 1. Introduction

Gastric cancer is the fourth most common type of cancer and the second leading cause of cancer death [1]. The incidence of this malignancy differs substantially by geographic region, indicating that modifiable lifestyle factors, especially dietary factors may influence the development of this disease [2].

Coffee is one of the most widely consumed beverages globally, and its potential role in the development of many chronic diseases, including various types of cancer has received considerable attention over the past several decades [3,4]. How coffee consumption may modulate risk of gastric cancer, however, remains a subject open for investigation.

The association of coffee consumption with risk of gastric cancer has been investigated in a number of epidemiologic studies, with ambiguous findings [5,6,7,8,9,10,11,12,13,14,15,16,17]. A previous meta-analysis of published case-control and cohort studies suggested a null relation [5]. However, most studies included in that meta-analysis were retrospective case-control studies which are subject to biases. Moreover, only four out of the seven cohort studies included were adjusted for multi-variables. Therefore, possible confounding by other behavioral factors was not well investigated. This merits particular attention when examining the exposure such as coffee that has been found to been highly correlated with unhealthy factors such as smoking, heavy alcohol drinking, high body mass index and low physical activity, *etc.* [10,12], all of which may increase risk of gastric cancer [18,19,20,21].

Since the 2006 meta-analysis [5], 5 large prospective cohort studies [6,9,10,12,16] examining the association of coffee consumption with risk of gastric cancer have emerged, and the results continued to be inconsistent. Increased risk was reported in the Swedish Mammography Cohort Study [9] and the NIH-AARP Diet and Health Study [12] (restricted to gastric cardia cancer), and possible decreased risk was suggested in a Finnish cohort [6] (restricted to men) and in a cohort consisting of Singapore Chinese [16] (restricted to specific women, e.g., nonsmokers), and finally, a null relation was reported in another Swedish cohort [10]. In an attempt to further elucidate the relationship between coffee consumption and risk of gastric cancer, we conducted this updated meta-analysis of prospective studies.

## 2. Materials and Methods

### 2.1. Literature Search and Selection

We searched for potentially relevant publications published before June 2014 on PubMed and Embase databases using the search terms “coffee” or “caffeine” in combination with “gastric cancer” or “stomach cancer” or “cardia cancer”, with no language restrictions. We also carefully reviewed the reference lists of retrieved publications in order to identify any further studies. The title and abstract of the relevant studies were screened and then the full papers of potentially eligible studies were carefully accessed. To be included, the study had to meet the following criteria: (1) study design was prospective; (2) exposure of interest was coffee consumption; (3) outcome was gastric cancer; and (4) relative risks (RRs) with corresponding 95% confidence intervals (CIs) were available (either reported or could be estimated according to reported data).

### 2.2. Data Extraction

Two authors independently extracted data using a standardized data-collection form, and discussion would be performed if there were any disagreements. The following data were extracted from each included eligible study: the first author’s last name, year of publication, country of origin, years of follow-up, sex of subjects, number of cases and subjects, levels of consumption, range of consumption, RRs of gastric cancer with corresponding 95% CIs, and statistical adjustment in the analysis.

### 2.3. Data Synthesis and Analyses

The RRs reflecting the greatest degree of control for confounders were used in the meta-analysis. The summary risk estimates were obtained with the method of DerSimonian and Laird using the assumptions of the random-effects model that takes into account both within-study and between-study variations [22]. Statistical heterogeneity amongst studies was assessed with Q and *I*^2^ statistics [23]. For the Q statistic, a *P-*value of less than 0.1 was considered statistically significant heterogeneity. Potential publication bias was evaluated by use of Begg’s funnel plots and Egger’s regression asymmetry test [24].

Stratified analyses were performed for the following subgroups: geographic areas, length of follow-up, number of cases, sex, range of consumption, and adjustment for potential confounders. A sensitivity analysis was also conducted by omitting one study at each turn and pooling results from the remaining studies. Because the levels of consumption among individual studies differed substantially, A dose-response analysis using the method by Greenland and Longnecker was conducted [25]. Accordingly, study-specific slopes (linear trends) and 95% CIs were estimated from the natural logs of the RRs and CIs across categories of coffee consumption. Eligible studies were those studies that reported the number of cases and person-years and the risk estimates with their variance estimates for at least three quantitative exposure categories. All statistical analyses were performed using STATA software, version 11.0 (StataCorp., College Station, TX, USA).

## 3. Results

### 3.1. Study Characteristics

**Figure 1:**
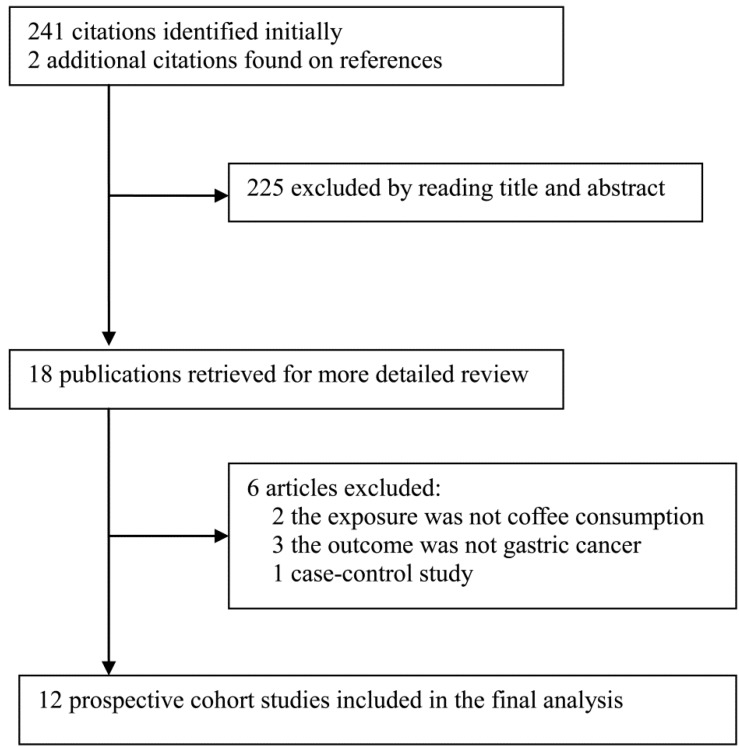
Flow chart showing process of literature search.

The flow chart of literature search is shown in Figure 1. Twelve studies [6,7,8,9,10,11,12,13,14,15,16,17] that evaluated the association of coffee consumption with risk of gastric cancer met the pre-defined inclusion criteria, and were included in the final analysis. The 12 studies contained a total of 2688 gastric cancer cases and 840,651 subjects. These studies were published between 1986 and 2014, and they were conducted in the United States (3 studies), Norway (2 studies), Sweden (2 studies), Japan (2 studies), Netherlands (1 study), Finland (1 study) and Singapore (1 study), respectively. The median or mean length of follow-up ranged from 5.4 years to 18 years; the number of cases ranged from 51 to 647; and the number of subjects ranged from 1525 to 481,562. The levels of coffee consumption, the risk estimates reported in the primary studies, as well as the variables adjusted in the analyses differed substantially. The study characteristics are summarized in Table 1.

### 3.2. Overall Analysis

**Figure 2:**
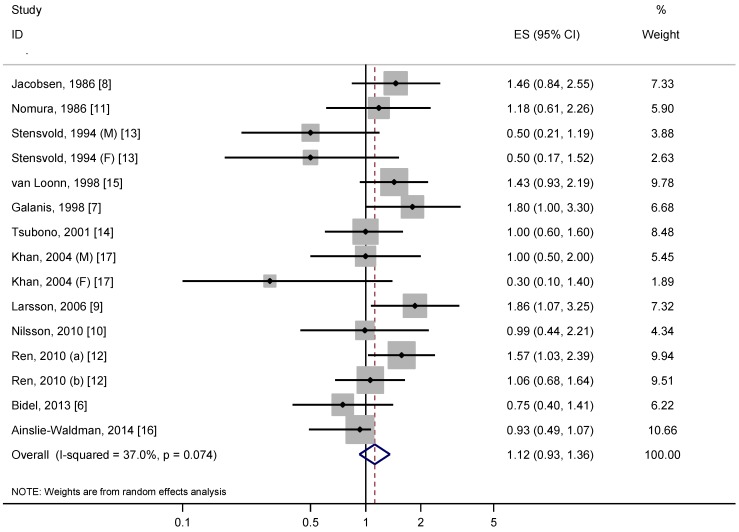
Relative risk of gastric cancer for the highest compared with the lowest categories of coffee consumption. (a) results for gastric cardia cancer; (b) results for gastric non-cardia cancer; M, male; F, female; RR, relative risk; CI, confidence interval.

A meta-analysis of the 12 studies suggested that high coffee consumption was not associated with risk of gastric cancer, with a summary RR of 1.12 (95% CI = 0.93–1.36), with moderate heterogeneity (*P* = 0.07, *I*^2^ = 37.0%) (Figure 2).

### 3.3. Stratified, Sensitivity and Dose-Response Analyses

The results of subgroup analysis stratified by geographic areas, length of follow-up, number of cases, sex, range of consumption, and adjustment for potential confounders are presented in Table 2. High consumption of coffee was found to be significantly associated with increased risk of gastric cancer in studies from the United States (RR = 1.35, 95% CI = 1.06–1.74), in studies with shorter duration of follow-up (RR = 1.24, 95% CI = 1.00–1.54), and in those studies without adjustment for smoking (RR = 1.48, 95% CI = 1.13–1.93). The study by Larsson *et al.* [9] was not adjusted for smoking, but the investigators found a similar result (RR = 1.21, 95% CI = 0.96–1.54) when restricting the analysis to a subset with information on smoking. However, treating this study as the one adjusted for smoking did not change the initial finding (RR = 1.38, 95% CI = 1.02–1.87) for those without adjustment and 1.04 (95% CI = 0.85–1.26) for those with adjustment. The sensitivity analysis carried out by omitting one study at each turn and pooling results from the remaining studies indicated that an exclusion of any single study did not materially change the main result.

**Table 1:** Characteristics of included prospective studies examining the association between coffee consumption and risk of gastric cancer.

Study	Country	Duration, Years	No. of Cases	No. of Subjects	Sex	Highest * vs.* Lowest Consumption	Range of Consumption	RR (95% CI)	Variables Adjusted for
Jacobsen, 1986 [8]	Norway	11.5	147	16555	M/F	≥7 *vs.* ≤2 cups/day	6.5 cups/day	1.46 (0.84–2.55) ^a^	Age, sex, and residence.
Nomura, 1986 [11]	USA	15	106	7355	M	≥5 *vs.* 0	5 cups/day	1.18 (0.61–2.26) ^b^	Age
Stensvold, 1994 [13]	Norway	10.1	80	42973	M/F	≥7 *vs.* ≤2 cups/day	4.5 cups/day	0.50 (0.21–1.19) (M) ^a^ 0.50 (0.17–1.52) (F) ^a^	Age, smoking, and residence.
van Loon, 1998 [15]	Netherlands	4.3	146	1525 (sub-cohort)	M	>4 *vs.* ≤4 cups/day	4 cups/day	1.43 (0.93–2.19) ^b^	-
Galanis, 1998 [7]	USA	14.8	108	11907	M/F	≥2 *vs.* 0 cups/day	3 cups/day	1.80 (1.00–3.30)	Age, sex, smoking (M), education and place of birth.
Tsubono, 2001 [14]	Japan	9	419	26311	M/F	≥3 *vs.* 0 cups/day	3.5 cups/day	1.00 (0.60–1.60)	Age, sex, smoking, consumption of tea, alcohol, rice, meat, vegetables, fruits and bean-past soup, and type of health insurance.
Khan, 2004 [17]	Japan	13.8 (M) 14.8 (F)	51	3155	M/F	≥several times/week *vs.* ≤several times/month	<1 cups/day	1.00 (0.50–2.00) (M) 0.30 (0.10–1.40) (F)	Age, smoking, health status (F), health education (F), and health screening (F).
Larsson, 2006 [9]	Sweden	15.7	160	61433	F	≥4 *vs.* ≤1 cups/day	4 cups/day	1.86 (1.07–3.25)	Age, calendar year, education, and consumption of tea andm alcohol.
Nilsson, 2010 [10]	Sweden	6	70	64603	M/F	≥4 *vs.* <1 cups/day	6.5 cups/day	0.99 (0.44–2.21)	Age, sex, BMI, smoking, education, and physical activity.
Ren, 2010 [12]	USA	5.4	455	481563	M/F	>3 *vs.* <1 cups/day	3 cups/day	1.57 (1.03–2.39) ^c^ 1.06 (0.68–1.64) ^d^	Age, sex, BMI, smoking, education, ethnicity, physical activity, and consumption of alcohol, fruits, vegetables, red meat, white meat and calories.
Bidel, 2013 [6]	Finland	18	299	60041	M/F	≥10 *vs.* 0 cups/day	11 cups/day	0.75 (0.40–1.41)	Age, sex, BMI, study year, education, smoking, physical activity, history of diabetes, and consumption of tea and alcohol.
Ainslie-Wal dman, 2014 [16]	Singapore	14.7	647	63257	M/F	≥4 cups/d *vs.* never/monthly	4.5 cups/day	0.93 (0.49–1.07)	Age, BMI, gender, interview year, dialect, education, smoking, intakes of caffeine and total energy intake.

BMI, body mass index; RR, relative risk; CI, confidence interval; M, male; F, female. ^a^ 95% confidence intervals were estimated according to reported original data; ^b^ relative risks and 95% confidence intervals were estimated according to reported original data; ^c^ results for gastric cardia cancer; ^d^ results for gastric non-cardia cancer.

### 3.4. Stratified, Sensitivity and Dose-Response Analyses

The results of subgroup analysis stratified by geographic areas, length of follow-up, number of cases, sex, range of consumption, and adjustment for potential confounders are presented in Table 2. High consumption of coffee was found to be significantly associated with increased risk of gastric cancer in studies from the United States (RR = 1.35, 95% CI = 1.06–1.74), in studies with shorter duration of follow-up (RR = 1.24, 95% CI = 1.00–1.54), and in those studies without adjustment for smoking (RR = 1.48, 95% CI = 1.13–1.93). The study by Larsson *et al.* [9] was not adjusted for smoking, but the investigators found a similar result (RR = 1.21, 95% CI = 0.96–1.54) when restricting the analysis to a subset with information on smoking. However, treating this study as the one adjusted for smoking did not change the initial finding (RR = 1.38, 95% CI = 1.02–1.87) for those without adjustment and 1.04 (95% CI = 0.85–1.26) for those with adjustment). The sensitivity analysis carried out by omitting one study at each turn and pooling results from the remaining studies indicated that an exclusion of any single study did not materially change the main result.

**Table 2:** Subgroup analysis for the association of coffee consumption with risk of gastric cancer.

Subgroups	*N*	RR (95% CI)	Heterogeneity Test	
			*P*	*I*^2^ (%)
**Areas**				
USA	3	**1.36 (1.06–1.74)**	0.44	0.0
Europe	6	1.08 (0.76–1.53)	0.06	50.9
Japan	3	0.92 (0.70–1.20)	0.40	0.0
**Duration of follow-up**				
≥10 years	8	1.02 (0.76–1.38)	0.03	50.4
<10 years	4	**1.24 (1.00–1.54)**	0.53	0.0
**No. of cases**				
≥200	4	1.07 (0.85–1.34)	0.29	19.7
<200	8	1.14 (0.85–1.53)	0.06	44.8
**Sex**				
Men	8	0.98 (0.70–1.36)	0.08	44.9
Women	6	1.04 (0.58–1.89)	0.06	53.1
**Range of consumption**				
≥5 cups/day	4	1.09 (0.79–1.51)	0.47	0.0
<5 cups/day	8	1.12 (0.88–1.42)	0.03	49.0
**Adjustment**				
Smoking, *yes*	8	0.99 (0.78–1.25)	0.10	38.2
*no*	4	**1.48 (1.13–1.93)**	0.77	0.0
Alcohol, *yes*	4	1.21 (0.90–1.62)	0.14	42.1
*no*	8	1.05 (0.81–1.37)	0.10	39.3
BMI, * yes*	4	1.07 (0.84–1.37)	0.29	19.0
*no*	8	1.14 (0.86–1.50)	0.05	46.1
Education, *yes*	6	1.22 (0.95–1.57)	0.12	40.1
*no*	6	0.99 (0.73–1.35)	0.12	39.3
Physical activity, *yes*	3	1.12 (0.82–1.54)	0.25	27.4
*no*	9	1.11 (0.87–1.43)	0.05	44.8
Dietary factors, *yes*	5	1.14 (0.89–1.47)	0.14	39.9
*no*	7	1.06 (0.78–1.45)	0.08	42.5

RR, relative risk; CI, confidence interval.

The dose-response analysis of nine eligible studies [6,7,9,10,11,12,13,14,16] conferred a RR of 1.03 (95% CI = 0.95–1.13) for an increase in coffee consumption of 2 cups/day, with moderate heterogeneity (*P* = 0.07, *I*^2^ = 41.4%) (Figure S1). The RR for an increase of 4 and 6 cups/day was 1.07 (95% CI = 0.90–1.27) and 1.11 (95% CI = 0.86–1.44), respectively.

### 3.5. Publication Bias

**Figure 3:**
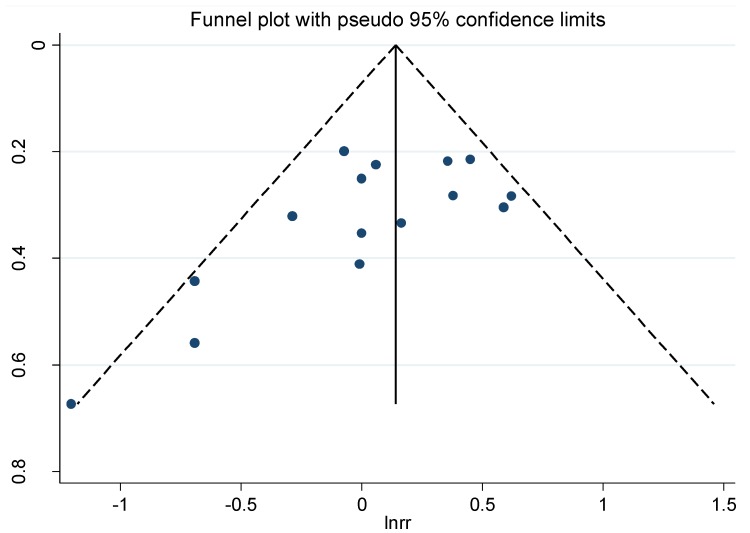
Begg’s funnel plot with pseudo-95% confidence limits for the RR of gastric cancer and coffee consumption (highest compared with lowest category of consumption).

In the overall meta-analysis, there was some evidence of publication bias according to the Egger’s test (*P* = 0.03), and the Begg’s funnel plots also showed some asymmetry (Figure 3).

## 4. Discussion

This meta-analysis quantitatively investigated the relation between coffee consumption and risk of gastric cancer, based on 12 prospective cohort studies involving a total of 2688 gastric cancer cases and 840,651 participants. The overall results suggested that coffee consumption was not associated with increased risk of gastric cancer. Our findings confirmed the results from the previous meta-analysis. Since 5 additional prospective studies consisting of more than 1630 cases and 0.7 million participants were available after the 2006 analysis, and all these studies had reported results adjusted for multi-variables, we were able to investigate the potential impacts of confounders on this association.

Significant increased risk of gastric cancer associated with high coffee consumption was observed in some analyses stratified by various pre-defined factors. These results, however, should be treated with caution because of the issue on inadequate adjustment for the major risk factor of gastric cancer. The strongest positive association between coffee consumption and gastric cancer was found in the four studies without adjustment for smoking; whereas in the remaining studies with adjustment, the association was null. Smoking has been consistently reported as the most important behavioral risk factor for gastric cancer [18]. Therefore, the possibility that smoking is responsible for the observed positive association merits further considerations because of the strong correlation between coffee consumption and cigarette smoking [10,12]. This possibility was somewhat supported by the results from Singapore Chinese Health Study [16], in which daily coffee consumption significantly decreased gastric cancer risk in non-smokers, and non-significantly increased the risk in smokers. Despite taking into account the number of cigarettes smoked, the possibility of confounding by smoking remains because of the relatively rough measurement of smoking.

Apart from smoking, other unmeasured confounders also merit discussions. Coffee consumption tends to be related to unhealthy behaviors, such as a high-salt diet that have been reported to increase gastric cancer risk [26]. Thus, not adjusting for salt intake (which was the case in all studies included) may exaggerate any harm of coffee; conversely, there is also evidence that coffee consumption decreases risk of diabetes, a potential risk factor for gastric cancer [27]. Thus, a failure to account for baseline diabetes status (which was the case in all except for one study [6]) may have attenuated any positive association towards a null.

Regarding sub-sites of gastric cancer, a significant positive association of coffee consumption with gastric cardia cancer (RR = 1.57, 95% CI = 1.03–2.39), and a null association with gastric non-cardia cancer (RR = 1.06, 95% CI = 0.68–1.64) was observed in the NIH-AARP study; but in the Singapore Chinese Health Study [16], the positive association with gastric cardia cancer was not confirmed (RR = 0.78, 95% CI = 0.46–1.33), and there was a trend towards reduced risk of gastric non-cardia cancer in women (RR = 0.68, 95% CI = 0.46–1.01). Only the Singapore Chinese Health Study [16] had taken into account the influence of helicobacter pylori (H. pylori) infection, and the results were similar before and after adjustment for H. pylori. There was one study [10] that considered filtered and boiled coffee separately, and no difference was found. There was no study that investigated the association by histological types of gastric cancer.

Potential mechanisms whereby coffee may modulate cancer risk have been extensively studied, with both protective and adverse effects proposed. Coffee is a complex mixture of hundreds of chemicals, among which caffeine and polyphenols are suspected to affect cancer risk. Coffee is rich in phenolic compounds that have been found to inhibit several carcinogens [28]. Experimental evidence demonstrates that phenolic compounds have anti-genotoxic and -oxidant properties [29,30], and can also suppress cancer growth through anti-estrogenic pathways or mitochondrial toxicity [31,32]. The findings for caffeine have been equivocal. Laboratory and animal studies suggest that caffeine can both promote and suppress the development of mammary tumors [33].

The strengths of this meta-analysis, in addition to the inclusion of large number of cases that enhanced the statistical power of the study, the prospective design of all included studies also minimized the risk of selection bias and information bias, both of which are a common concern in case-control studies in which exposure was determined after the diagnosis of diseases. One may question the validity of information about coffee consumption in case-control studies because cases may have differentially recalled their diet intakes, or have reduced their coffee consumption due to the precursor conditions of gastric cancer (e.g., pain in the abdomen).

There are also several limitations that should be pointed out. As is mentioned above, the first one is the concern of insufficient adjustment. The second concern is non-differential misclassification of exposure resulting from measurement error, which may have attenuated any true relation. Third, none of the individual studies considered differences in preparation methods, which may affect the concentration of different compounds in coffee [34]. Finally, publication bias is another concern because this meta-analysis is based on published studies. We observed some evidence of publication bias, indicating that the overall strength of the association observed may still be an overestimation.

## 5. Conclusions

In conclusion, findings from this meta-analysis of prospective studies suggest that coffee consumption is not associated with risk of gastric cancer. Future prospective studies carefully accounting for potential confounders for gastric cancer are warranted.

## Supplementary Files

Supplementary File 1
