# Supplementary material for: Coffee Consumption and Risk of Gastric Cancer: A Large Updated Meta-Analysis of Prospective Studies
Source: Nutrients. 2014 Sep 18;6(9):3734–46. doi: 10.3390/nu6093734 (PMC4179186; doi:10.3390/nu6093734)
Supplement: Supplementary File 1 [file nutrients-06-03734-s001.docx]

**Supplementary Information**

**Figure S1**. Results of dose-response meta-analysis for an increase in coffee consumption of 2 cups/day.

References

1. Nomura, A.; Heilbrun, L.K.; Stemmermann, G.N. Prospective study of coffee consumption and the risk of cancer. *J. Natl. Cancer Inst*. **1986**, *76*, 587–590.
2. Stensvold, I.; Jacobsen, B.K. Coffee and cancer: A prospective study of 43,000 norwegian men and women, *Cancer Causes Control* **1994**, *5*, 401–408.
3. Galanis, D.J.; Kolonel, L.N.; Lee, J.; Nomura, A. Intakes of selected foods and beverages and the incidence of gastric cancer among the japanese residents of hawaii: A prospective study. *Int. J. Epidemiol.* **1998**, *27*, 173–180.
4. Tsubono, Y.; Nishino, Y.; Komatsu, S.; Hsieh, C.C.; Kanemura, S.; Tsuji, I.; Nakatsuka, H.;
   Fukao, A.; Satoh, H.; Hisamichi, S.; *et al*. Green tea and the risk of gastric cancer in japan. *N. Engl. J. Med*. **2001**, *344*, 632–636.
5. Larsson, S.C.; Giovannucci, E.; Wolk, A. Coffee consumption and stomach cancer risk in a cohort of swedish women. *Int. J. Cancer* **2006**, *119*, 2186–2189.
6. Bidel, S.; Hu, G.; Jousilahti, P.; Pukkala, E.; Hakulinen, T.; Tuomilehto, J. Coffee consumption and risk of gastric and pancreatic cancer—A prospective cohort study. *Int. J. Cancer* **2013**, *132*, 1651–1659.
7. Ren, J.S.; Freedman, N.D.; Kamangar, F.; Dawsey, S.M.; Hollenbeck, A.R.; Schatzkin, A.;
   Abnet, C.C. Tea, coffee, carbonated soft drinks and upper gastrointestinal tract cancer risk in a large united states prospective cohort study. *Eur. J. Cancer* **2010**, *46*, 1873–1881.
8. Bidel, S.; Hu, G.; Jousilahti, P.; Pukkala, E.; Hakulinen, T.; Tuomilehto, J. Coffee consumption and risk of gastric and pancreatic cancer—A prospective cohort study. *Int. J. Cancer* **2013**, *132*, 1651–1659.
9. Ainslie-Waldman, C.E.; Koh, W.P.; Jin, A.; Yeoh, K.G.; Zhu, F.; Wang, R.; Yuan, J.M.;
   Butler, L.M. Coffee intake and gastric cancer risk: The singapore chinese health study. *Cancer Epidemiol. Biomark. Prev.* **2014**, *23*, 638–647.

© 2014 by the authors; licensee MDPI, Basel, Switzerland. This article is an open access article distributed under the terms and conditions of the Creative Commons Attribution license (http://creativecommons.org/licenses/by/3.0/).
